# Distinct serum endocannabinoid profiles in treatment-naïve Han Chinese children with ADHD: a case–control pilot study

**DOI:** 10.3389/fneur.2025.1715342

**Published:** 2026-01-12

**Authors:** Wenjuan Liao, Xiaobin Tan, Jinhai Lin, Yuchen Wu, Qi Guo, Qucheng Huang, Longhe Yang, Yan Peng

**Affiliations:** 1 Department of Pharmacy, First Affiliated Hospital of Gannan Medical University, Ganzhou, China; 2 First Clinical Medical College, Gannan Medical University, Ganzhou, China; 3 Technical Innovation Center for Utilization of Marine Biological Resources, Third Institute of Oceanography, Ministry of Natural Resources, Xiamen, China

**Keywords:** ADHD, endocannabinoids, oleoylethanolamide, palmitoylethanolamide, 2-arachidonoylglycerol, anandamide, SNAP-IV, oppositional defiant disorder

## Abstract

**Objective:**

Although peripheral endocannabinoid system (ECS) signatures have been implicated in ADHD among European and American populations, evidence from Asian pediatric cohorts remains scarce. This study quantified serum concentrations of four major endocannabinoids—anandamide (AEA), 2-arachidonoylglycerol (2-AG), oleoylethanolamide (OEA), and palmitoylethanolamide (PEA)—in treatment-naïve Han Chinese children with ADHD and examined their associations with symptom severity as measured by the SNAP-IV scale.

**Methods:**

This cross-sectional study enrolled 22 children with ADHD (aged 6–12 years, diagnosed per DSM-5, IQ > 70) and 25 healthy controls (aged 6–12 years). Serum endocannabinoids were quantified via liquid chromatography–tandem mass spectrometry (LC–MS/MS). Symptom severity was assessed with SNAP-IV subscales [inattention, hyperactivity/impulsivity, oppositional defiant disorder (OD)]. Group comparisons used *t*-tests, and correlations were analyzed with Spearman’s rank coefficient.

**Results:**

Children with ADHD showed significantly lower OEA (1.21 ± 0.14 ng/mL vs. 1.65 ± 0.16 ng/mL) and PEA (0.69 ± 0.06 ng/mL vs. 0.86 ± 0.05 ng/mL) levels, higher 2-AG (1.94 ± 0.08 ng/mL vs. 1.72 ± 0.017 ng/mL, *p* = 0.001), and unchanged AEA (0.33 ± 0.05 ng/mL vs. 0.36 ± 0.05 ng/mL) compared to controls. In the ADHD group, OEA negatively correlated with OD scores (rs = −0.461, *p* = 0.031), but not with inattention or hyperactivity/impulsivity; other endocannabinoids showed no significant correlations.

**Conclusion:**

Selective ECS alterations in pediatric ADHD, particularly reduced OEA/PEA and elevated 2-AG with OEA’s link to OD symptoms, may highlight potential blood-based biomarkers for diagnosis and monitoring, warranting further research into ECS-targeted therapies.

## 1 Introduction

Attention-Deficit/Hyperactivity Disorder (ADHD) is the most prevalent neurodevelopmental disorder in childhood, affecting approximately 5–7% of children worldwide. In China, large-scale studies and meta-analyses confirm a similar prevalence, with estimates ranging from 5.6 to 6.5% among children and adolescents (1, 2). ADHD is a multifactorial and etiologically heterogeneous disorder influenced by genetic, epigenetic, neurobiological, and environmental factors, with no single determinant being causally sufficient (3–5). Diagnostic approaches have transitioned from behavioral ratings to objective multimodal assessments, incorporating neuroimaging, neurochemistry, and genomics (6, 7). Structural MRI reveals prefrontal-striatal circuit maturational delays (8, 9), while positron emission tomography (PET) studies show aberrant dopamine transporter densities (10). Treatment innovations have progressed beyond stimulants to include atomoxetine and viloxazine, but 20–30% of children show partial response or intolerable side effects such as insomnia, appetite loss, gastrointestinal symptoms, mood changes, and, rarely, cardiovascular events (11–13). Crucially, longitudinal studies confirm that early neuroanatomical differences predict long-term outcomes (14, 15), emphasizing the need for preventive strategies. However, clinical judgment and multi-informant reports remain central, as no single objective test is yet definitive (16, 17).

The endocannabinoid system (ECS), comprising CB1/CB2 receptors, endocannabinoids [anandamide (AEA), 2-AG, oleoylethanolamide (OEA), and palmitoylethanolamide (PEA)], and metabolic enzymes (FAAH/MAGL), regulates synaptic plasticity, dopamine release, and emotional processing neurocircuitries (18, 19). Preclinical models consistently demonstrate ECS dysregulation in ADHD-like phenotypes: CB2 knockout mice exhibit hyperactivity (20), while FAAH deficiency enhances anandamide signaling and reduces impulsivity (21). In humans, genome-wide studies identify ADHD risk alleles in ECS-associated genes, including FAAH rs2295633 (22). Research suggests that CB1 cannabinoid receptors in the striatum play a modulatory role in neural circuits relevant to ADHD, but direct evidence in children with ADHD is limited. Most insights come from animal models and developmental studies (23, 24). These molecular alterations disrupt excitatory-inhibitory balance, hyperactivating reward pathways (25) and impairing top-down executive control. Peripheral blood profiling has emerged as a minimally invasive window into central ECS dysfunction. A recent study showed that individuals with substance use disorder (SUD) and comorbid ADHD exhibit lower plasma concentrations of 2-AG, 2-LG, and AEA, while PEA is elevated compared to SUD patients without ADHD and healthy controls. Machine learning models identified AEA, OEA, PEA, and SEA as key biomarkers for distinguishing between healthy controls, SUD without ADHD, and SUD with ADHD, achieving moderate to good diagnostic performance (accuracy: 72.1%, AUC: 0.77) (26). Although derived from adults with comorbid SUD, these data prompted us to examine whether similar ECS shifts exist in treatment-naïve children. In neurodevelopmental disorders like Tourette syndrome (often comorbid with ADHD), CSF levels of 2-AG, AEA, and PEA are elevated, and 2-AG levels correlate with ADHD symptom severity (27). This suggests a compensatory or pathophysiological role for endocannabinoids in ADHD-related symptoms. Experimental models show that AEA and PEA depletion impairs learning, memory, and emotional responses, while 2-AG is more involved in short-term synaptic plasticity (28). These findings support the idea that endocannabinoid signaling modulates cognitive and emotional processes relevant to ADHD.

Despite growing evidence supporting ECS dysregulation in ADHD, existing data are derived predominantly from adult or adolescent populations with comorbid substance-use disorders and are limited to European and American cohorts. Systematic information on peripheral ECS profiles in Asian children with ADHD remains absent. To address this gap, the present study quantified serum concentrations of key ECS-related lipids (2-AG, AEA, PEA, OEA) in treatment-naïve Han Chinese children aged 6–12 years with DSM-5 ADHD and examined their correlations with clinician-rated symptom severity (SNAP-IV). Our findings provide the first ethnicity-specific reference values for childhood ADHD, validate the utility of blood ECS biomarkers across diverse populations, and open new avenues for early, objective diagnosis and personalized cannabinoid-targeted interventions in pediatric mental health.

## 2 Materials and methods

### 2.1 Study population

A total of 47 children aged 6–12 years were recruited, comprising 22 children diagnosed with ADHD (ADHD group) and 25 healthy children (control group). The ADHD group included children referred for initial evaluation, while the control group consisted of children attending routine health check-ups. After screening based on these criteria, the ADHD group consisted of 22 children (18 boys, 4 girls; age range 6–12 years, mean age 8.6 ± 1.6 years; total IQ score 91.4 ± 5.7). The control group included 25 children (15 boys, 10 girls; age range 6–12 years, mean age 7.8 ± 1.7 years; total IQ score 88.3 ± 5.4). Baseline characteristics, including age, gender, IQ scores, body weight, and BMI showed no statistically significant differences between groups (*p* > 0.05; Table 1). Dietary habits were assessed via a brief parental questionnaire, excluding children with known nutritional deficiencies or extreme diets (e.g., vegan or high-fat diets) that could confound ECS measurements. All participants’ legal guardians provided written informed consent, following the principles outlined in the Declaration of Helsinki.

**Table 1:** Baseline characteristics of the study groups.

Parameter	ADHD (*n* = 22)	Control (*n* = 25)	*p*-value
Age (years, mean ± SD)	8.6 ± 1.6	7.8 ± 1.7	ns^a^
Gender (*n*, male/female)	22, 18/4	25, 15/10	–
WISC-IV Total IQ Score (mean ± SD)	91.4 ± 5.7	88.3 ± 5.4	ns
Height (m, mean ± SD)	1.31 ± 0.10	1.27 ± 0.12	ns
Body Weight (kg, mean ± SD)	28.8 ± 7.0	27.0 ± 6.7	ns
BMI	16.5 ± 1.4	16.4 ± 1.0	ns

a Non-significant values in statistical analysis (ns).

### 2.2 Inclusion criteria

For the ADHD group: (1) Diagnosis of ADHD confirmed according to DSM-5 criteria and SNAP-IV by two independent specialists (a pediatric psychiatrist and a clinical psychologist) (29); (2) Age 6–12 years; (3) Intelligence quotient (IQ) > 70 on the Wechsler Intelligence Scale for Children, Fourth Edition (WISC-IV) (30), indicating cognitive function comparable to age-matched norms; (4) First-time diagnosis with no prior exposure to psychotropic medications.

For the control group: (1) healthy children attending routine pediatric check-ups; (2) no medication uses in the preceding 6 months that could influence biomarker levels; (3) IQ > 70 on the WISC-IV.

### 2.3 Exclusion criteria

Exclusion criteria for both group: (1) comorbid psychiatric disorders such as schizophrenia; (2) concurrent organic diseases affecting other organs; (3) history of brain injury, hemorrhage, or other neurological conditions; (4) any prior use of cannabis or exposure to second-hand cannabis smoke (assessed via parental interview); (5) documented dietary deficiencies, including but not limited to essential fatty acids or vitamins that could impact ECS function.

### 2.4 Ethical considerations

This study was conducted in accordance with the ethical guidelines of the Declaration of Helsinki (1964) and received approval from the Institutional Ethics Committee of our hospital (Resolution No. LLSC-2024-341). Parents/guardians and children (where age-appropriate) were fully informed about the study’s aims, procedures, risks, and benefits. Written informed consent was obtained from all guardians, and verbal assent was secured from children.

### 2.5 Sample collection

Peripheral venous blood samples (3 mL) were collected between 8:00 a.m. and 10:00 a.m. after an overnight fast to minimize diurnal variability. Samples were drawn into tubes, centrifuged at 3000 × *g* for 10 min at 4 °C, and serum was aliquoted and stored at −80 °C until analysis. No invasive procedures beyond venipuncture were involved.

### 2.6 Quantification of endocannabinoids

Serum levels of anandamide (AEA), 2-arachidonoylglycerol (2-AG), oleoylethanolamide (OEA), and palmitoylethanolamide (PEA) were quantified by liquid chromatography–tandem mass spectrometry (LC–MS/MS) on an ACQUITY UPLC I-Class System coupled to a Xevo TQ-MS mass spectrometer (Waters, Milford, MA, USA). Extraction was performed as previous described (31–33). Briefly, 200 μL serum was spiked with 200 μL methanol containing 1 nmol 10Z-heptadecenoylethanolamide (Avanti Polar Lipids, USA) as internal standards. After vortex-mixing for 30 s, 1 mL chloroform (CHCl₃, LC–MS grade) was added, and the mixture was vortexed again (1 min) and centrifuged (12,000 × *g*, 15 min, 4 °C). The lower organic layer was transferred to a clean glass tube, dried under a gentle nitrogen stream, and reconstituted in 100 μL methanol/CHCl₃ (90:10, v/v).

Chromatographic separation was achieved on an Agilent ZORBAX Eclipse Plus C18 column (2.1 × 50 mm, 2.5 μm) maintained at 40 °C. The binary gradient (flow rate 0.4 mL min^−1^) consisted of (A) 0.1% (v/v) formic acid in water and (B) 0.1% formic acid in acetonitrile. The programme was: 0–2.50 min 70%A → 5%A, 2.50–4.50 min held at 5%A, 4.50–5.50 min 5%A → 70%A, followed by 0.5 min re-equilibration at 70%A (total run time 6.0 min). Injection volume was 5 μL and the autosampler tray was kept at 4 °C.

Mass-spectrometric detection was carried out in positive electrospray ionisation (ESI^+^) multiple-reaction monitoring (MRM) mode. Source parameters were: capillary 3.0 kV, desolvation temperature 500 °C, desolvation gas 1,000 L h^−1^, cone gas 150 L h^−1^. Collision energies and cone voltages were optimized for each analyte. Molecular ions (precursor → product) were monitored at m/z 348.2 → 62.1 (AEA), 379.3 → 287.3 (2-AG), 326.3 → 62.1 (OEA), 300.1 → 62.1 (PEA) and 312.1 → 62.1 (10Z-heptadecenoylethanolamide, IS). Calibration curves (0.1–100 ng mL^−1^) exhibited r^2^ ≥ 0.99. The lower limit of detection (LLOD) was 0.1 ng mL^−1^ for all analytes.

### 2.7 Statistical analysis

Data were analyzed using Graphpad prism 9 (San Diego, California, USA). Continuous variables are presented as mean ± standard deviation (SD) in table or mean ± standard error of the mean (SEM) in figure. Between-group comparisons used independent t-tests for normally distributed data. Correlations between SNAP-IV subscale scores (inattention, hyperactivity/impulsivity, OD) and serum endocannabinoid levels were evaluated using Spearman’s rank correlation coefficient (*r*_s_) in the ADHD group, given the non-parametric nature of symptom scores. Statistical significance was set at *p* < 0.05 (two-tailed).

## 3 Results

The final sample included 47 children (22 in the ADHD group, 25 in the control group). Baseline demographics showed no significant differences in age, gender distribution, or IQ scores (Table 1). Anthropometric parameters (height, weight, BMI score) were comparable between groups (*p* > 0.05; Table 1), confirming group homogeneity.

**Figure 1:**
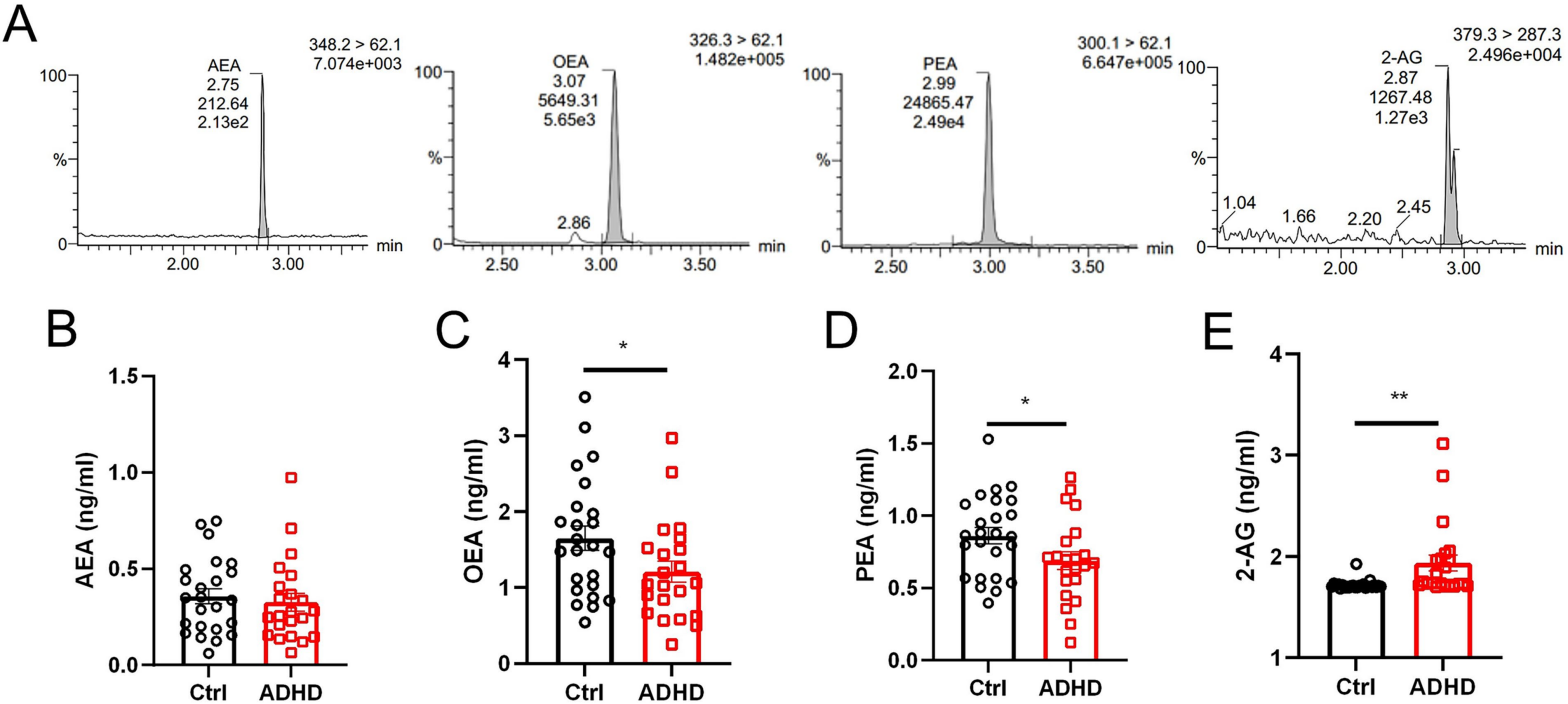
Serum endocannabinoids in Ctrl and ADHD determined by LC–MS/MS. **(A)** Representative MRM chromatograms and group data for serum endocannabinoids. Concentrations (ng mL^−1^) of **(B)** AEA, **(C)** OEA, **(D)** PEA, **(E)** 2-AG measured in Ctrl (black circles) and ADHD (red square) cohorts. Data expressed as mean ± SEM; *t*-test, *p < 0.05, **p < 0.01.

Serum endocannabinoid levels are showed in Figure 1. Compared to the control group, children in the ADHD group exhibited significantly lower levels of OEA (mean 1.21 ± 0.14 ng/mL vs. 1.65 ± 0.16 ng/mL) and PEA (mean 0.69 ± 0.06 ng/mL vs. 0.86 ± 0.05 ng/mL). In contrast, 2-AG levels were significantly elevated in the ADHD group (mean 1.94 ± 0.08 ng/mL vs. 1.72 ± 0.017 ng/mL). No significant difference was observed in AEA levels between groups (mean 0.33 ± 0.05 ng/mL in ADHD vs. 0.36 ± 0.04 ng/mL in controls).

**Figure 2:**
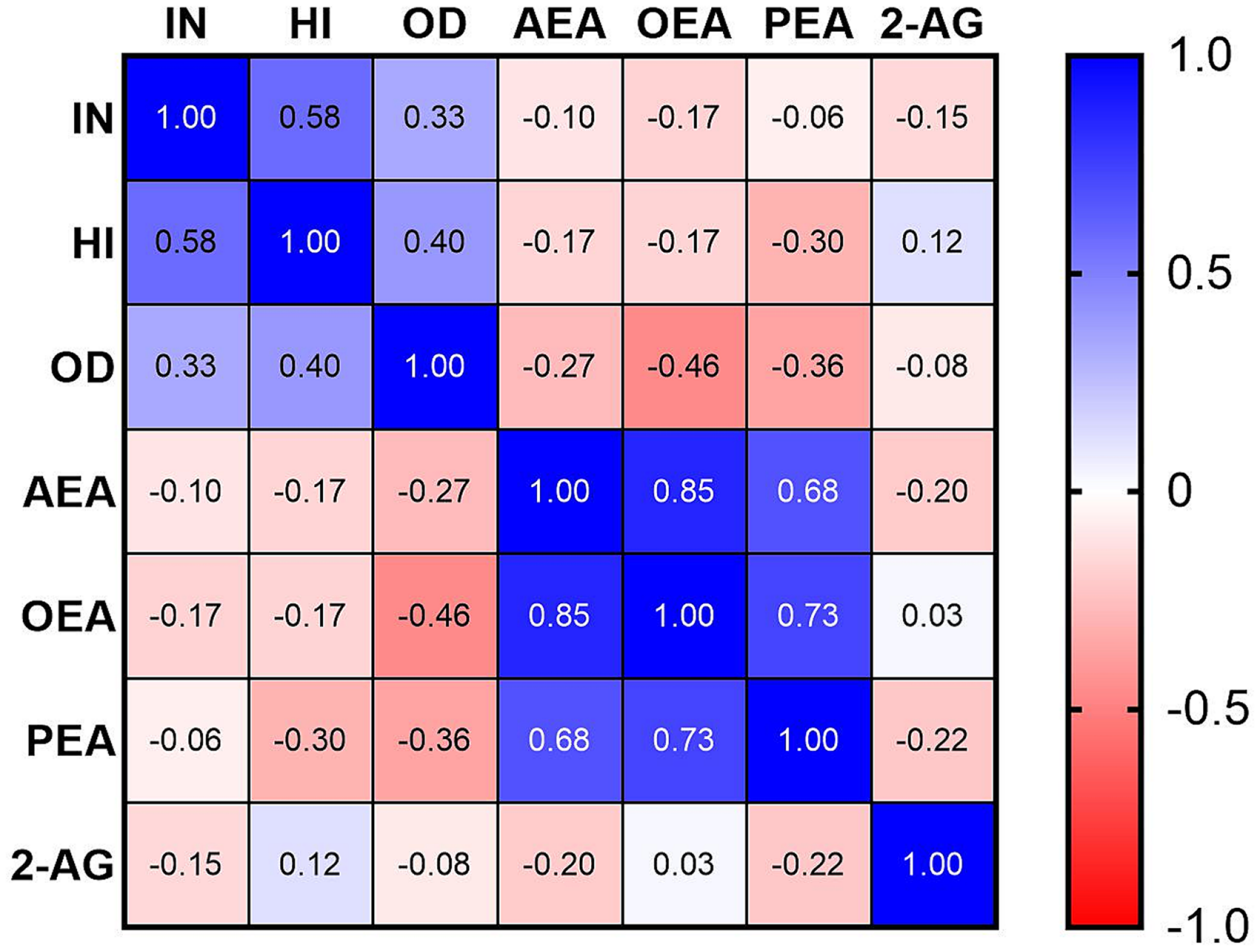
Spearman’s correlations between serum endocannabinoid levels and SNAP-IV subscale scores in the ADHD group.

Mean SNAP-IV subscale scores in the ADHD group were 1.66 ± 0.38 for inattention (IN), 1.24 ± 0.61 for hyperactivity/impulsivity (HI), and 1.04 ± 0.62 for oppositional defiant (OD). Correlation analyses within the ADHD group (Table 2, Figure 2) showed a significant negative correlation between serum OEA levels and OD scores (*r*_s_ = −0.461, *p* = 0.031). No significant correlations were found between OEA and IN (*r*_s_ = −0.175, *p* = 0.437) or HI (*r*_s_ = −0.170, *p* = 0.45). Similarly, PEA, 2-AG, and AEA levels did not correlate significantly with any SNAP-IV subscales (all |*r*_s_| < 0.36, *p* > 0.05). These findings indicate selective alterations in the endocannabinoid system profile in pediatric ADHD, with reductions in OEA and PEA alongside an increase in 2-AG, while AEA remains unchanged, and a specific inverse relationship between OEA and OD symptoms.

**Table 2:** Spearman’s correlations between serum endocannabinoid levels and SNAP-IV subscale scores in the ADHD group.

ADHD group	SNAP-IV (score)		
	Inattention (IN)	Hyperactivity-impulsivity (HI)	Oppositional defiant (OD)
AEA	−0.103	−0.168	−0.27
OEA	−0.175	−0.170	**−0.461** ^*^
PEA	−0.063	−0.296	−0.363
2-AG	−0.150	0.125	−0.082

*p-value <0.05.

## 4 Discussion

The present study demonstrates selective alterations in serum endocannabinoid levels among treatment-naïve children with ADHD, characterized by significantly reduced OEA and PEA, elevated 2-AG, and unchanged AEA compared to healthy controls. Furthermore, OEA levels exhibited a moderate negative correlation with oppositional defiant disorder (OD) symptom scores on the SNAP-IV scale, independent of inattention or hyperactivity/impulsivity subscales. These findings support our hypothesis that ECS dysregulation contributes to the neurodevelopmental pathophysiology of ADHD, potentially influencing behavioral and executive functions through modulation of dopaminergic and glutamatergic pathways.

Interpreting these results in the context of prior research reveals both consistencies and divergences in endocannabinoid profiles across neurodevelopmental and neuropsychiatric disorders. Elevated 2-AG levels in our pediatric ADHD cohort resonate with observations in adult ADHD populations, where plasma 2-AG concentrations are similarly increased, suggesting a persistent ECS hyperactivity that may exacerbate impulsivity and reward-seeking behaviors (34). This elevation could reflect compensatory mechanisms to counteract dopaminergic deficits, as 2-AG is known to enhance synaptic plasticity in prefrontal and striatal regions implicated in ADHD (18). However, our finding of unchanged AEA contrasts with some studies reporting increased AEA in peripheral blood mononuclear cells of children with ADHD, attributed to reduced fatty acid amide hydrolase (FAAH)-mediated degradation (35), and elevated plasma AEA in adults with ADHD (34). These discrepancies may stem from methodological differences, such as sample type (serum vs. plasma or cellular isolates), age-specific ECS maturation, or the exclusion of comorbid conditions in our study, highlighting the need for standardized quantification protocols like LC–MS/MS across age groups.

The selective elevation of 2-AG without corresponding changes in AEA may also reflect differential regulatory pathways within the ECS. While both are endogenous ligands for CB1 receptors, 2-AG is primarily synthesized on-demand via diacylglycerol lipase (DAGL) in response to synaptic activity and degraded by monoacylglycerol lipase (MAGL), whereas AEA is produced through N-acyl phosphatidylethanolamine phospholipase D (NAPE-PLD) and hydrolyzed by fatty acid amide hydrolase (FAAH) (1). Repeated stress or hyperdopaminergic states (as seen in ADHD models) can elevate 2-AG levels, enhancing the capacity for 2-AG-mediated short-term synaptic suppression, particularly at inhibitory synapses (36). 2-AG’s modulation of dopamine is more robust than AEA’s, making it a primary regulator of dopamine-driven behaviors in these circuits (37). Conversely, stable AEA levels might suggest that its role in longer-term emotional regulation and anti-anxiety effects is less perturbed in treatment-naïve pediatric ADHD, though this could vary with age or comorbidity. These distinctions underscore symptom-specific ECS involvement, where 2-AG elevations may preferentially link to hyperactivity/impulsivity, warranting targeted MAGL inhibitors as potential therapeutics (38).

Regarding OEA and PEA reductions, our results represent a novel observation in pediatric ADHD, differing from the limited direct evidence in this disorder but paralleling patterns in related neurodevelopmental conditions. For instance, in children with autism spectrum disorder (ASD)—which shares substantial comorbidity with ADHD (up to 50–70% overlap)—serum or plasma levels of OEA, PEA, and AEA are consistently lower than in healthy controls, potentially linked to impaired social–emotional processing and anti-inflammatory deficits (39, 40). These NAE congeners (OEA and PEA) exert anti-inflammatory and neuroprotective effects via peroxisome proliferator-activated receptor-alpha (PPAR-α) activation, and their depletion in ADHD may exacerbate neuroinflammation or oxidative stress, contributing to symptom severity (41–44). In contrast, studies on other neuropsychiatric disorders, such as major depressive disorder (MDD) in women, show no significant alterations in hair concentrations of endocannabinoids and N-acylethanolamines (including PEA and OEA) (45), underscoring disorder-specific ECS profiles. The negative correlation between OEA and OD symptoms in our ADHD group further suggests that diminished OEA signaling may selectively disrupt inhibitory control over oppositional behaviors, possibly through reduced modulation of prefrontal-amygdala circuits (19).

In a broader context, these findings implicate the ECS as a convergence point for genetic, environmental, and neurobiological factors in ADHD etiology. Environmental exposures like lead or vitamin D deficiency may interact with ECS components to amplify dysregulation, as preclinical models show that such toxins alter endocannabinoid tone in developing brains (46). The implications extend to clinical practice: selective ECS alterations could serve as non-invasive serum biomarkers for ADHD subtyping, enabling early identification of at-risk children with prominent OD features and guiding personalized interventions. Moreover, the therapeutic potential of ECS modulation—such as FAAH inhibitors to boost AEA/OEA/PEA or CB1 agonists to normalize 2-AG—warrants exploration, given anecdotal reports of cannabinoid benefits in ADHD symptom management (47). However, caution is advised due to risks of cannabis use disorder in ADHD populations, where self-medication rates are high (48).

This study has several limitations. First, the small sample size (*n* = 47) limits generalizability and statistical power for subgroup analyses, though it serves as a pilot to inform larger studies. Second, while we controlled for key confounders like age, IQ, and basic diet, unmeasured variables such as genetic polymorphisms in ECS genes (e.g., CNR1 or FAAH) or subtle environmental exposures could influence results. Third, serum measurements provide a peripheral snapshot and may not fully reflect central ECS activity; future studies incorporating neuroimaging or CSF sampling could bridge this gap. Fourth, the cross-sectional design precludes causal inferences; longitudinal tracking of ECS changes with symptom progression or treatment would be valuable. To address these limitations, future research should prioritize larger, longitudinal cohorts to track ECS changes from childhood through adulthood, incorporating neuroimaging to correlate serum levels with brain ECS activity. Interventional trials targeting NAE elevation (e.g., via PEA supplementation) could validate therapeutic efficacy, while investigating gene–environment interactions (e.g., CNR1 polymorphisms) may elucidate why some ADHD subtypes exhibit divergent endocannabinoid profiles. Overall, our study advances understanding of ECS involvement in pediatric ADHD, paving the way for mechanism-based diagnostics and treatments.

## Data Availability

The original contributions presented in the study are included in the article/supplementary material, further inquiries can be directed to the corresponding authors.
